# A three layered repair of a large perineal hernia: case report and review of the literature

**DOI:** 10.1186/s40792-023-01636-5

**Published:** 2023-04-12

**Authors:** Hagai Soback, Lauren Lahav, Rotem Franko, Shmuel Avital

**Affiliations:** Surgery B Department, Meir Medical Center, Tchernichovsky St. 59, Kfar Saba, Israel

**Keywords:** APR, eAPR, Perineal hernia, Biological mesh

## Abstract

**Background:**

A symptomatic perineal hernia is an uncommon complication after abdominoperineal resection (APR). Repairs of such hernias can be achieved by usage of autologous flaps, synthetic mesh, or biologic mesh, which reduce bowel adhesions. Studies have shown that prophylactic repair of the pelvic floor with biologic mesh during APR, can reduce the incidence of perineal hernia.

**Case presentation:**

A 71-year-old woman, after extended APR (eAPR) with primary closure of pelvic floor with absorbable mesh, presented to our outpatient clinic with a symptomatic, extensive perineal hernia. The patient underwent repair of the perineal hernia using a synthetic mesh and a bilateral gluteal flap procedure. In post operative care, signs of surgical site infection and a fluid collection demonstrated in a CT-scan, compelled a surgical drainage. A clear fluid negative for bacterial growth was drained and antibiotic treatment was initiated. After drainage, surgical site showed signs of significant improvement and patient was eventually discharged.

**Conclusion:**

The rise in reported incidence of perineal hernia after eAPR coupled with the scarcity of data regarding the preferable repair technique suggests that there is a significant need for further prospective comparative studies.

## Background

Symptomatic perineal hernia is considered a rare complication after abdominoperineal resection (APR) [1]. The reported incidence of symptomatic perineal hernia is estimated to occur in less than 1% of the cases after APR [2]. Symptoms usually include pain and discomfort when standing or sitting, urinary problems, intestinal obstruction, and perineal skin breakdown [3]. Risk factors for perineal herniation after pelvic resection include preoperative chemoradiotherapy and smoking among others [4]. Repair of perineal hernia can be accomplished using mesh including the use of biological meshes, which are better suited for contaminated fields and can decrease bowel adhesions when compared to synthetic mesh [5].

The study by Musters et al. on patients undergoing extralevator abdominoperineal resection (eAPR) with primary closure compared with patients closed with a biological mesh showed that wound healing after preoperative radio-therapy did not improve when a biological mesh was used. Yet, a significantly lower 1-year perineal hernia rate was observed in the biological mesh closure group [6].

## Case presentation

Here we present a case of a 71-year-old woman, with a history of rectal adenocarcinoma. Initially treated with a local excision of the tumor, that was categorized T2N0 according to imaging done prior to the surgery (MRI and transrectal ultrasound). 6 days after the local excision, patient presented with signs of pelvic sepsis due to perforation of the surgical wound. Consequently, a diverting sigmoid-loop-colostomy and debridement was performed. The colostomy was closed 6 months later.

Almost 3 years after the colostomy closure the patient was diagnosed with a local recurrence and underwent a laparoscopic extralevator abdominoperineal resection (eAPR) with end colostomy. During the eAPR the pelvic floor was prophilactly closed using a biological, fast absorbable mesh in order to prevent future perineal herniation (GORE® BIO-A® Tissue Reinforcement, biosynthetic web scaffold, 67% polyglycolic acid, 33% trimethylene carbonate. Flagstaff, AZ, USA). Pathology samples revealed mucinous 2 cm diameter adenocarcinoma with circumferential radial margins of 6 mm and negative for metastasis in 14 lymph nodes.

After the eAPR, the patient was treated with chemoradiotherapy, with 27 rounds of radiation and oral treatment of capecitabine (Xeloda, Genentech, CA, US). During oncological follow up, the patient has no evidence of recurrence (2 years) as of writing this article.

**Fig. 1:**
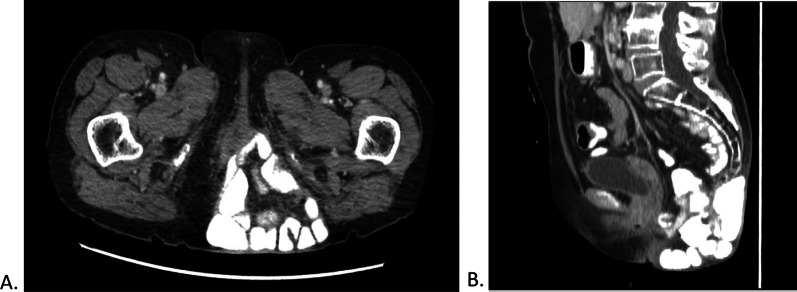
**A** CT-scan of pelvic outlet showing herniation of multiple, small intestine loops, with contrast material. Axial view. **B** Extent of herniation shown from a midsagittal view

About 2 years after eAPR, the patient presented to our outpatient clinic with complaints of a noticeable bulge in her perineum, which could be painful during standing and ambulating. Physical examination demonstrated a large, irreducible herniation of the perineal area. A CT scan was performed, showing a very large perineal hernia containing small intestine, with a hernia orifice measuring 4.7 cm and 7.5 cm in the sagittal and transverse aspects respectively (Fig. 1A, B). Due to her complaints and radiological findings, a corrective operation was indicated and performed. A three layer repair was considered appropriate considering the large and substantial cavity that would remain. The repair should withstand the high pressure of sitting and movement, and provide comfort in a seated position, especially considering the patient's complaints of discomfort. Because of significant thinning of the perineal skin, and in order to further fortify the closure, it was also decided to close the skin in a “vest over pants” formation.

**Fig. 2:**
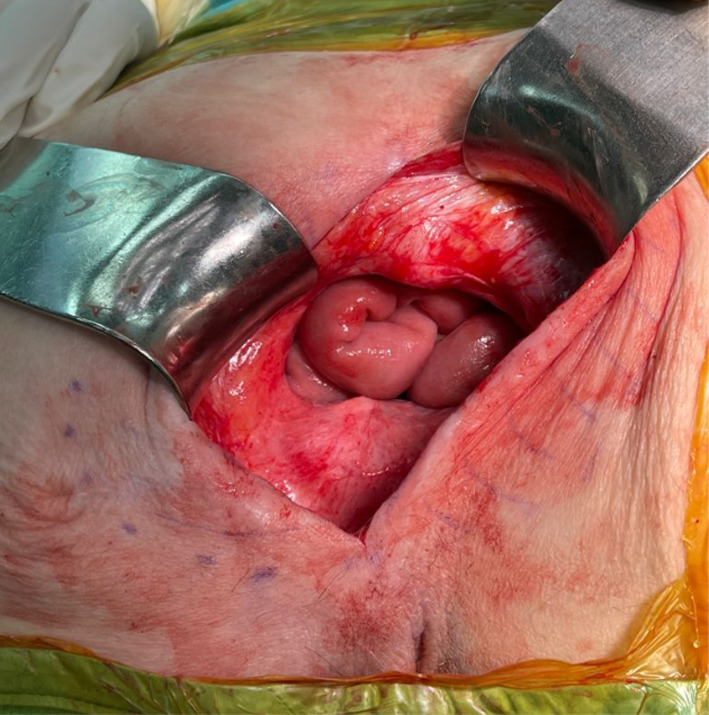
Herniation sac as seen during operation

The patient was placed in a jack knife position throughout the surgery. After herniation sac was identified and reduced (Fig. 2), a three layered repair was performed together with surgeons from the plastic surgery department—a 15 × 20 cm synthetic Composite mesh was used to close and reinforce the pelvic floor (15 × 20 cm Symbotex™ Composite Mesh by Medtronic, Minneapolis, MN, USA). The mesh was sutured to the coccyx, pubis, ischium, and cervix. The plastic surgeons assisted in forming a bilateral gluteal muscle flaps, approximately 3 cm from each side, which were sutured to each other, end to end, with interrupted 2–0 Vicryl sutures. The skin was then closed in a “vest over pants” formation after the underlying skin was de-epithelialized.

In post-operative-day 11, due to suspected surgical site infections, A CT-scan was performed showing a fluid collection. The fluid was drained in the operating theater. A drain was placed in the remaining space and antibiotic treatment was initiated. The surgical site showed signs of clinical improvement after the drainage, and fluid culture was negative for bacterial growth. The patient was eventually discharged on post-operative-day 23 after the herniation repair.

**Fig. 3:**
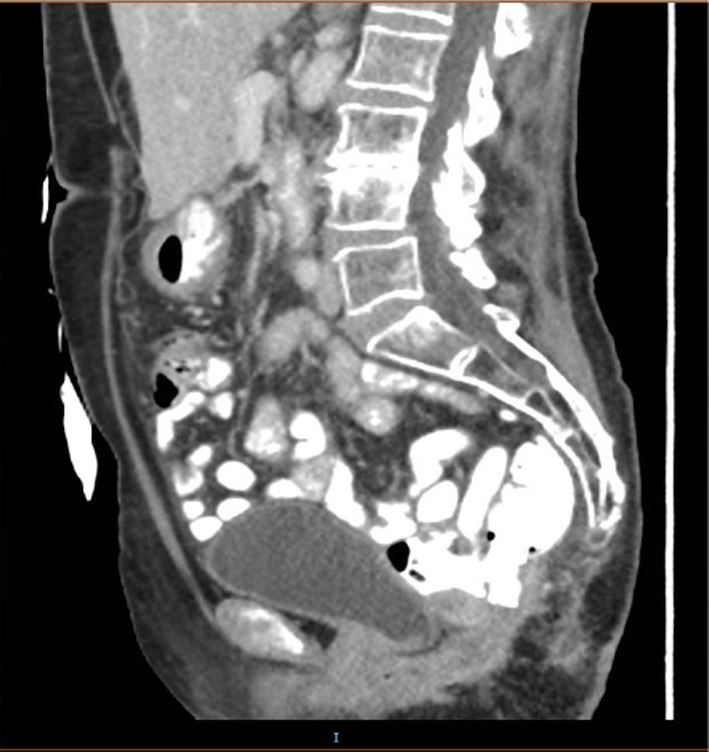
A follow up CT-scan showing results of the hernia repair, 2 months after the operation

**Fig. 4:**
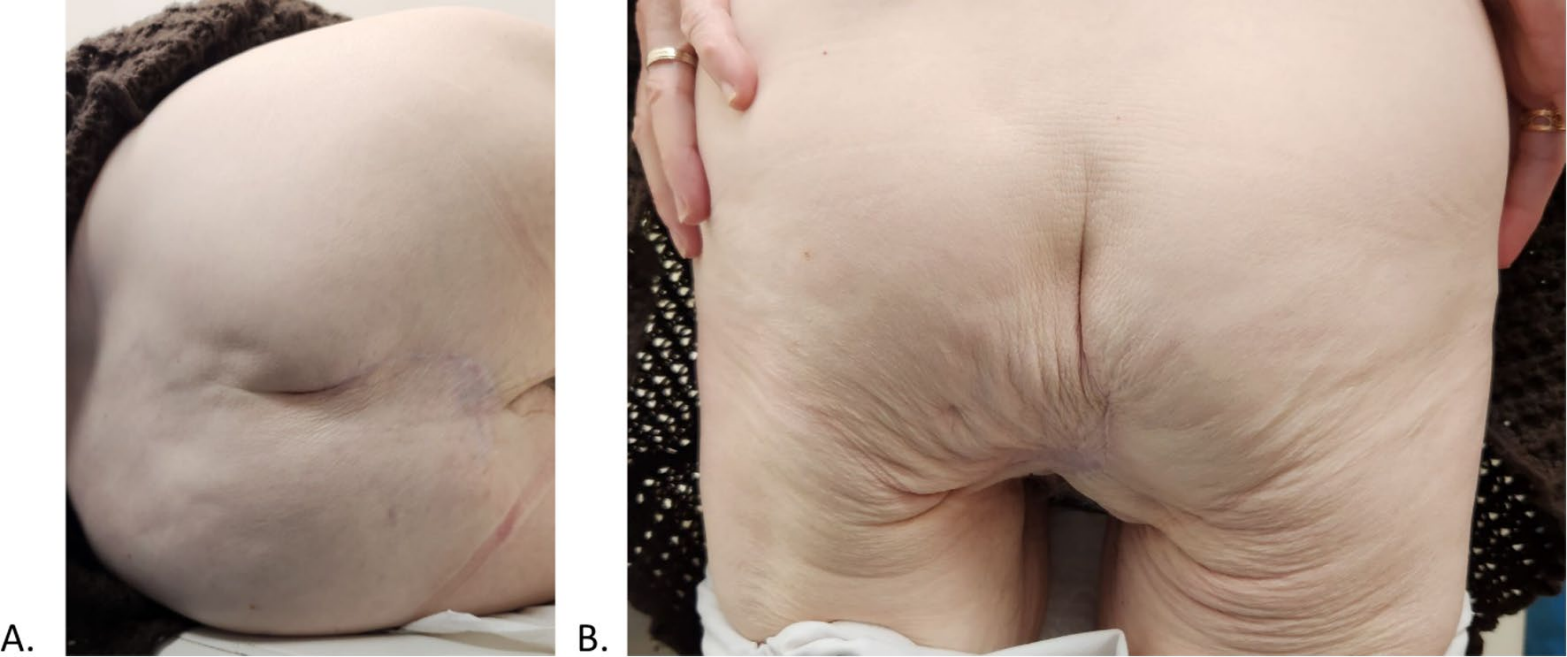
**A** Patient lying in a left lateral decubitus showing no signs of herniation. **B** Patient standing showing no signs of herniation

In a follow up imaging study 2 months after discharge, a CT-scan shows no recurrence of perineal herniation (Fig. 3). Follow up examination done a year after the operation, showed satisfying surgical results, and no sign of recurrence has been found in a physical examination (Fig. 4A, B). Yet the patient still reports some discomfort while sitting on hard surfaces.

## Discussion

Data suggesting the incidence of perineal hernia after perineal excision to be lower than 1% is mostly historic and mainly regards APR [4]. More recent studies showed the incidence of perineal hernia after eAPR reached 26% regardless if primary closure was performed with biological mesh, myocutaneous flap or by a simple suture of the perineal skin and closure of the peritoneum [7]. Data of different secondary repair methods of perineal hernia are also scarce and of poor quality without a general agreement on which operative strategy is optimal for repair of perineal hernia after eAPR [8]. Other studies have shown some advantage to closing the perineum with biological mesh, yet not by a large margin [6]. Across all recent studies mentioned, there seems to be an agreement that incidence of perineal hernia is higher than previously thought [6–8]. Possibly due to the increased, more extensive resection done in eAPR to yield better oncological results. Therefore, further prospective studies appear warranted.

Literature discussing the possible benefits of a prophylactic repair during eAPR are even more scarce. While some have suggested such surgical protocols using a slow absorbable mesh [9], no others have been described that we are aware of.

In the case presented here, the female patient who underwent primary repair with a fast absorbable mesh during her eAPR, still developed a very large symptomatic perineal hernia about 2 years after the resection. In her repair operation both synthetic mesh and myocutaneous flap were utilized to prevent a recurrence of the herniation. It is important to note that even though the patient showed signs of wound complications after surgery utilizing a synthetic mesh, drainage and antibiotic treatment achieved successful wound healing.

There are no reports of primary repair during eAPR with a synthetic mesh most probably due to the contaminated nature of the procedure. A recent study regarding a single stage repair of contaminated ventral hernias with synthetic mesh compared with biological mesh demonstrated that while both meshes had similar safety profiles, synthetic mesh yielded superior results over biologic mesh with regard to 2-year hernia recurrence risk [10].

Considering the reported rise in the incidence of perineal hernia after eAPR, a primary closure of the pelvic floor with mesh or autologous flap might be justified. The absence of a clear evidence based recommended technique hinders efforts to potentially avoid or, subsequently, repair them.

## Conclusion

In order to prevent or treat perineal hernia after eAPR, further prospective studies are much needed. Until such data are made available, patient selection is key. The rise in reported perineal hernia after eAPR also warrants the study of primary closure techniques.

## Data Availability

The data used for this case report is available from the corresponding author upon request.

## Abbreviations

APR: Abdominoperineal resection
eAPR: Extended abdominoperineal resection
MRI: Magnetic resonance imaging
TRUS: Transrectal ultrasound
CT: Computed tomography
